# Effectiveness of Osteopathic Manipulative Treatment in Treating Symptoms of Irritable Bowel Syndrome: A Literature Review

**DOI:** 10.7759/cureus.42393

**Published:** 2023-07-24

**Authors:** Cheri Lotfi, Jesse Blair, Angela Jumrukovska, Mackenzie Grubb, Emily Glidden, James Toldi

**Affiliations:** 1 Osteopathic Medicine, Lake Erie College of Osteopathic Medicine, Bradenton, USA; 2 Sports Medicine, Lake Erie College of Osteopathic Medicine, Bradenton, USA

**Keywords:** ibs, irritable bowel syndrome, abdominal bloating, abdominal pain, visceral osteopathy, osteopathic manipulative treatment (omt)

## Abstract

Irritable bowel syndrome (IBS) is a common gastrointestinal disorder that affects a patient for their entire life. Effective treatments for IBS are scarce, leading to an increased interest in alternative treatments such as osteopathic manipulative treatment (OMT). OMT uses hands-on treatment to reduce pain through various methods. By focusing on visceral techniques, OMT can restore autonomic homeostasis and increase lymphatic flow. This literature review aims to investigate the efficacy of visceral OMT in reducing the severity of IBS symptoms. Five primary research studies were evaluated in this analysis. The concluding results show that visceral OMT effectively reduces the symptoms of IBS and improves patients' quality of life. Therefore, OMT should be considered an alternative therapy for treating IBS.

## Introduction and background

Irritable bowel syndrome (IBS) is a common gastrointestinal (GI) condition that affects approximately 5-20% of the population and has an annual incidence of 196-260 per 100,000 [1]. It is more prevalent in women than in men, and while it may affect all age groups, it often presents in the third or fourth decade of life. Genetics, early life trauma, and chronic stress seem to increase an individual’s susceptibility, but the underlying mechanism of IBS remains unclear [1]. Food, bacteria, inflammation, and environmental conditions can have an influence on luminal function. All of these factors should be taken into consideration as it is suspected that IBS is of multifactorial origin. Symptoms can range from tolerable to severe, which may have an extensive impact on an individual's quality of life. These symptoms include continuous or intermittent abdominal pain, bloating, flatulence, and bowel habit changes. Traditional treatment approaches, including lactose reduction, fiber supplementation, bulking agents, laxatives, antispasmodics, antibiotics, and antidepressants, provide minimal therapeutic value [2]. The growing interest in enhancing the management of IBS has led to a focus on alternative treatment options like osteopathic manipulative medicine.

IBS can be classified as a “gut-brain axis” disorder because the processing of visceral stimuli is altered and sub-optimal in function, leading to the majority of symptoms patients experience [3]. Therefore, physicians have proposed osteopathic manipulative treatment (OMT) to reduce IBS symptoms by restoring homeostatic balance, normalizing autonomics, and improving lymphatic flow. The addition of visceral techniques to the OMT of IBS has been studied and aims to provide a direct influence on the intestinal flow and function of the colonic tract [4]. This includes the use of treatments such as colonic stimulation and mesenteric release, which directly promote intestinal health and have been considered for patients with constipation-predominant symptoms. 

This review aims to answer the effectiveness and safety of the use of osteopathic treatment in reducing the severity and symptoms of IBS in patients.

This article was previously presented as a poster at the 2023 Florida Osteopathic Medical Association Research Poster Competition on February 3rd, 2023, the LECOM Bradenton Interprofessional Research Day on April 27, 2023, and at the 2023 FMA Poster Symposium on July 29, 2023 (Appendices).

## Review

Methods

**Figure 1:**
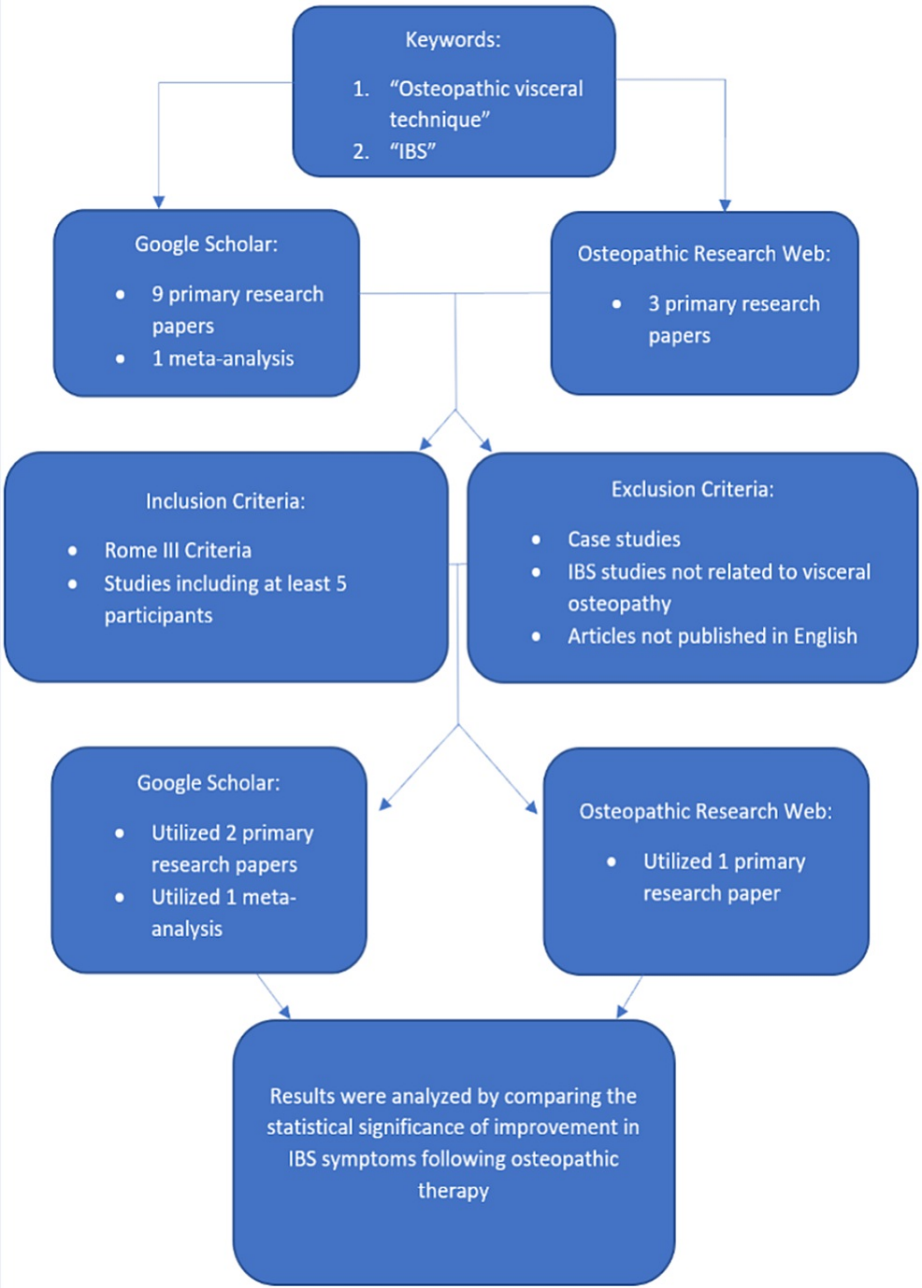
Meta-analysis methods This outlines the inclusion and exclusion criteria used in our research.

Primary research studies and meta-analyses examining the effect of OMT on the symptom severity of IBS were identified using academic search engines, including Google Scholar and Osteopathic Research Web by E.G. in August 2022. Keywords “osteopathic visceral techniques” and “Irritable Bowel Syndrome” were utilized in each search engine. Nine primary research studies and one meta-analysis were found using Google Scholar, and three primary research studies were found using the Osteopathic Research Web. These were all reviewed by C.L., J.B., A.J., M.G., E.G., and J.T. The articles most relevant to the research question were determined based on described inclusion and exclusion criteria. Our study included only those research papers that met two criteria: First, they utilized the Rome III Criteria, and second, they had a minimum of five participants. Case studies and articles published in languages other than English were excluded from our study. IBS studies that did not relate to OMT were also excluded from our study. Overall, two primary research studies and one meta-analysis were included from Google Scholar, and one primary research study was included from the Osteopathic Research Web. We came across a fifth study, Hundscheid et al., which was referenced in the research study by Attali et al. Based on the inclusion and exclusion criteria discussed, we included Hundscheid et al. in our analysis. Results were analyzed by comparing the statistical significance of improvement in IBS symptoms following OMT. A flow diagram of included studies is detailed in Figure 1.

Results

Four out of the five studies were randomized controlled studies, and the fifth study was an intervention study. Each study used different methods to analyze outcomes. Attali et al. and Florance et al. analyzed IBS symptom severity, Steiner used the Irritable Bowel Syndrome Quality of Life Instrument (IBS-QOL) and IBS symptom severity (IBS-SS) questionnaires, and Hundscheid et al. and Stiedl et al. used score sheets such as the Likert/visual analog (VAS) scale [3-7]. Additionally, each research study utilized different OMT techniques. Attali et al. and Steiner used visceral manipulation while Florance et al. used a combination of indirect and direct OMT techniques [3-5]. Hundscheid et al. used multiple OMT techniques and Stiedl et al. custom-tailored the OMT techniques to each individual [6,7].

Attali et al., Florance et al., Hundscheid et al., and Stiedl et al. demonstrated statistical significance with P values less than 0.01, as seen in Table 1 (4-7). In Steiner's study, the IBS-QOL questionnaire revealed statistical significance with a P value of approximately 0.015, whereas the IBS-SS questionnaire showed a P value of 0.02 for total daily symptom improvement and a P value of 0.015 for individually most severe symptom improvement [3]. When comparing the outcome results, Attali et al. not only showed a decrease in rectal sensitivity but also demonstrated short-term and long-term relief from abdominal distension and pain, lasting up to one year [4]. Steiner demonstrated a decrease in daily IBS-related symptoms [3]. Florance et al. began to show a decrease in IBS symptom severity in the experimental group on day seven after OMT [5]. Hundscheid et al. revealed OMT significantly improved overall IBS symptoms among the osteopathy group with a P value less than 0.006 [6]. Stiedl et al. stated that mean pain levels according to the VAS dropped tremendously in the OMT group (64.5 to 12.9) when compared to the group that received no OMT (63.7 to 49.7) - almost a four times difference in the reduction of pain levels [7]. The study demonstrated that OMT leads to an improvement in constipation intensity among participants [7]. Important characteristics and findings for all included studies are displayed in Table 1.

**Table 1:** Research study outlines Brief outlines of all five research articles included in this study are compiled here [3-7]. IBS: irritable bowel syndrome; VAS: visual analog scale; IBS-SS: IBS symptom severity; IBS-QOL: IBS quality of life

	"Treatment of refractory irritable bowel syndrome with visceral osteopathy: Short-term and long-term results of a randomized trial" Attali et al.	"The Effect of Visceral Osteopathic Treatment on Irritable Bowel Syndrome" Steiner	"Osteopathy improves the severity of irritable bowel syndrome: a pilot randomized sham-controlled study" Florance et al.	"Treatment of irritable bowel syndrome with osteopathy: results of a randomized controlled pilot study" Hundscheid et al.	"Osteopathy as a promising short-term strategy for irritable bowel syndrome: A randomized controlled trial" Stiedl et al.
Study Objective	"Evaluate the effectiveness of visceral osteopathy for IBS."	"Investigate the effect of visceral manipulation techniques on the quality of life and severity of symptoms of subjects suffering from IBS."	"Evaluate the effect of osteopathy on the severity of IBS in a randomized sham-controlled trial."	"Evaluate the effects of osteopathic treatments for IBS."	To understand how a predefined osteopathic treatment can affect the symptoms of IBS.
Study Design	Randomized, crossover placebo-controlled study	Intervention study	Randomized sham-controlled study	Randomized controlled trial	Randomized sham-controlled study
Number of Participants	31	8	30	20	61
Osteopathic Technique(s)	Visceral manipulation and sacral techniques	Visceral manipulation	Combination of direct and indirect techniques	Multiple techniques	Custom-tailored with multiple techniques
Outcome Measurement	Reporting of IBS symptom severity following treatment	IBS-QOL and IBS-SS questionnaires	IBS Severity Scale	IBS symptom score sheet using the 5-point Likert scale/IBS-QOL questionnaire	VAS/Likert scales for frequency and intensity of symptoms
Statistical Significance	IBS symptoms improved among the experimental group (P < 0.001)	IBS-QOL (P = 0.015)/IBS-SS total daily symptom improvement (P = 0.02)/IBS-SS individually most severe symptom improvement (P = 0.015)	IBS symptoms improved among the experimental group (P = 0.01)	IBS symptoms improved among the experimental group (P < 0.006)/IBS-QOL (P < 0.009)	IBS symptoms improved among the experimental group (P < 0.01)
Outcome Results	Statistically significant decrease in short-term and long-term abdominal distension and pain. Additionally, a decrease in rectal sensitivity was shown.	Statistically significant decrease in daily IBS-related symptoms	Statistically significant decrease in IBS symptom severity among the experimental group on the 7th day following osteopathic treatment	Statistically significant improvement in overall symptoms with osteopathic therapy	Statistically significant improvement in the intensity of constipation among participants treated with osteopathic therapy compared to the sham group.

Discussion

A combined analysis of all research studies indicates that OMT has the ability to ease IBS symptoms. Attali et al. discovered that visceral OMT alleviated constipation, diarrhea, abdominal distension, rectal hypersensitivity, and abdominal pain [4]. Steiner showed that visceral manipulation significantly decreased daily IBS symptoms in patients [3]. Florance et al. concluded that standardized OMT effectively decreased the abdominal pain associated with IBS in the short-term duration [5]. This study also suggested OMT sessions should be conducted at least once a month to maximize the clinical benefit [5]. Hundscheid et al. recognized that OMT patients had a better quality of life with respect to the symptom score [6]. Overall, Stiedl et al. concluded that OMT reduced the symptoms of IBS, including abdominal pain, constipation, diarrhea, and general well-being [7].

There are several theories on how visceral manipulation can improve the symptoms of those with IBS. After somatic manipulation treatment, some physiologic properties improve. These include increased fluid dynamics and nutrition to supply tissue, relaxing smooth muscle in fascia and ligaments, increased blood flow, and improved lymphatic drainage [3]. Additionally, the OMT benefits are amplified if more than one body region (i.e., the abdomen and sacrum) are manipulated. Treating IBS patients with OMT on the abdomen and surrounding viscera helps normalize the blood supply, lymphatic flow, and autonomic balance to restore normal motility. This highlights the osteopathic tenet that the body’s structure and function are reciprocally interrelated. Overall, visceral manipulation can help alleviate IBS symptoms due to improving the function of these properties. These research articles validate that OMT is effective in the treatment of IBS symptoms and can be integrated into standardized care.

Attali et. al, Florance et al., and Hundscheid et al. reported no adverse side effects for groups treated with OMT [4-6]. Steiner and Stiedl et al. omitted any report on safety [3,7].

Limitations

Overall, osteopathic treatment use for IBS symptoms has shown a great response, with no known adverse effects. Even so, there are some limitations in this review on osteopathy for the treatment of IBS. The studies reviewed used different visceral and soft tissue techniques as opposed to standardizing a single technique. The techniques that were conducted varied in length of time and frequency, which may have contributed to the greater degree of symptom resolution in some sample groups. Researchers utilized varying numbers of osteopathic physicians for treatment. The studies employed various severity scores and did not compare different types of severity scores in the treatment of IBS. Lastly, our sample size was small. A larger sample size would have increased the power of this study and decreased the margin of error.

## Conclusions

This review demonstrates a correlation between the benefits of visceral osteopathy and a reduction in the severity of IBS symptoms. The OMT performed did not appear to cause any side effects. Therefore, OMT may be considered a safe alternative or adjunct in the treatment of patients with IBS. However, more research is needed due to a limited number of randomized controlled studies relating to the effectiveness of OMT in alleviating IBS symptoms. Future studies will help demonstrate a stronger causal positive relationship between the benefits of visceral OMT and the reduction of IBS symptoms.

## Figures and Tables

**Figure 2:**
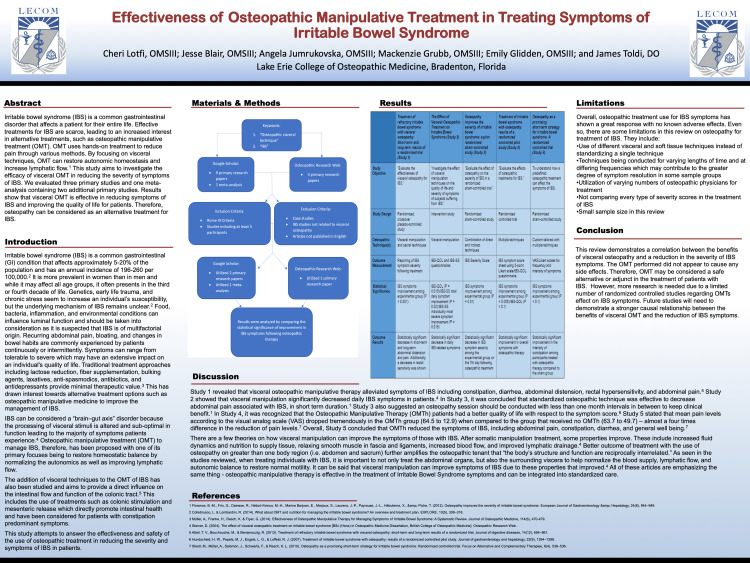
Poster presentation
